# Substitutions in the *Escherichia coli* RNA polymerase inhibitor T7 Gp2 that allow inhibition of transcription when the primary interaction interface between Gp2 and RNA polymerase becomes compromised

**DOI:** 10.1099/mic.0.062547-0

**Published:** 2012-11

**Authors:** Andrey Shadrin, Carol Sheppard, Konstantin Severinov, Steve Matthews, Sivaramesh Wigneshweraraj

**Affiliations:** 1 Section of Microbiology and MRC Centre for Molecular Bacteriology and Infection, Imperial College London, London SW7 2AZ, UK; 2 Waksman Institute for Microbiology and Department of Molecular Biology and Biochemistry, Rutgers, The State University of New Jersey, Piscataway, NJ 08854, USA; 3 Institute of Molecular Genetics, Russian Academy of Sciences, Moscow 123182, Russia; 4 Institute of Gene Biology, Russian Academy of Sciences, Moscow 119334, Russia

## Abstract

The *Escherichia coli*-infecting bacteriophage T7 encodes a 7 kDa protein, called Gp2, which is a potent inhibitor of the host RNA polymerase (RNAp). Gp2 is essential for T7 phage development. The interaction site for Gp2 on the *E. coli* RNAp is the β′ jaw domain, which is part of the DNA binding channel. The binding of Gp2 to the β′ jaw antagonizes several steps associated with interactions between the RNAp and promoter DNA, leading to inhibition of transcription at the open promoter complex formation step. In the structure of the complex formed between Gp2 and a fragment of the β′ jaw, amino acid residues in the β3 strand of Gp2 contribute to the primary interaction interface with the β′ jaw. The 7009 *E. coli* strain is resistant to T7 because it carries a charge reversal point mutation in the β′ jaw that prevents Gp2 binding. However, a T7 phage encoding a mutant form of Gp2, called Gp2^β^, which carries triple amino acid substitutions E24K, F27Y and R56C, can productively infect this strain. By studying the molecular basis of inhibition of RNAp from the 7009 strain by Gp2^β^, we provide several lines of evidence that the E24K and F27Y substitutions facilitate an interaction with RNAp when the primary interaction interface with the β′ jaw is compromised. The proposed additional interaction interface between RNAp and Gp2 may contribute to the multipronged mechanism of transcription inhibition by Gp2.

## Introduction

Central to the regulation of bacterial transcription is the RNA polymerase (RNAp), the enzyme responsible for the synthesis of all RNA in bacteria. The bacterial RNAp is composed of a multisubunit catalytic core (subunit composition α_2_ββ′ω; abbreviated E) and a sigma (σ) factor subunit that determines the specificity of the RNAp-promoter interaction. In *Escherichia coli*, the σ^70^-containing RNAp (Eσ^70^) is responsible for the transcription of most genes during exponential growth (Haugen *et al.*, 2008). Transcription by Eσ^70^ begins with the formation of the initial Eσ^70^–promoter complex known as the closed promoter complex (RPc). The RPc is often very unstable and isomerizes, via several intermediates, to the transcriptionally proficient open promoter complex (RPo). In the RPo, ~15 base pairs of promoter DNA around the transcription start site are melted to form the ‘transcription bubble’ and the template DNA strand is positioned at the active centre of the RNAp. The catalytic β and β′ subunits of the RNAp define the main DNA binding channel (DBC), and the active centre where RNA synthesis takes place is located deep within the DBC. The double-stranded DNA immediately downstream of the active centre (dwDNA) interacts with a segment of the DBC called the downstream DBC (dwDBC), and this interaction is essential for the formation and stability of the RPo (Saecker *et al.*, 2011).

**Fig. 1.**
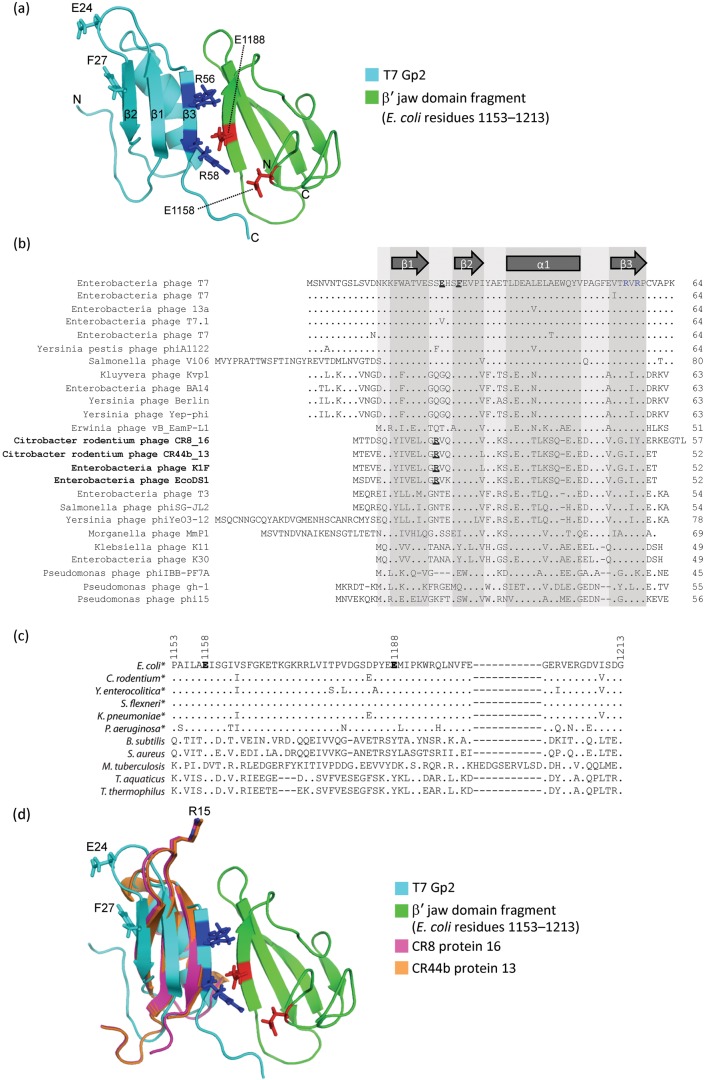
Sequence alignment analysis of T7 Gp2-like proteins. (a) Ribbon representation of the T7 Gp2–*E. coli* RNAp β′ jaw domain fragment (residues 1153–1213) complex. Gp2 is shown in cyan and the β′ jaw domain fragment is shown in green. The positions of the amino acids E24, F27 and R56 in T7 Gp2 are indicated along with the E1158 and E1188 residues in the β′ jaw domain. (b) Alignment of amino acid sequences from known Gp2-like proteins. The sequences are displayed in single-letter amino acid code, with the length of the sequence indicated on the far right. The localization of the β-strands and α-helix based on the structure of T7 Gp2 is indicated. The highly conserved R56 and R58 residues are highlighted in blue. The positions of amino acids residues E24 and F27 in T7 Gp2 are underlined and in bold type. The arginine residue, corresponding to T7 Gp2 amino acid residue S23 in Gp2-like proteins in phages CR8, CR44b, K1F and EcoDS1, is also underlined in bold type. (c) Alignment of the β′ jaw domain amino acid residues 1153–1213 from a representative set of host RNAps, with those infected by T7-like phages encoding Gp2-like proteins indicated by asterisks. Amino acid residues corresponding to E1158 and E1188 of the *E. coli* RNAp are shown in bold type. (d) As in (a), except that the structural models of Gp2-like proteins CR44b_13 (orange) and CR8_16 (pink) are superimposed and amino acid residue R15 is indicated. The structural models of the Gp2 homologues were calculated with swiss-model using NMR structures of T7 Gp2 as a template (Arnold *et al.*, 2006; Cámara *et al.*, 2010).

The RPo formation also represents an important regulatory step during transcription initiation, and thus the activity of the bacterial RNAp is controlled by an array of transcription regulatory factors, which repress, stimulate or modulate its ability to form the RPo (Browning & Busby, 2004). Most bacteriophages (phages) rely on the RNAp of their host bacteria for expression of their genes during the infection process. Therefore, RPo formation by the bacterial RNAp is also subjected to regulation by phage-encoded proteins through covalent RNAp modifications and modulation by RNAp-binding proteins (Nechaev & Severinov, 2003). T7 phage encodes a 7 kDa protein, called Gp2, which binds to a domain in the β′ subunit, called the ‘jaw’ domain, and potently inhibits RPo formation by the *E. coli* RNAp (Nechaev & Severinov, 1999). The β′ jaw domain is an integral component of the dwDBC and plays a major role during RPo formation and stable maintenance of the RPo (Ederth *et al.*, 2002, 2006). The biological role of T7 Gp2 is to ensure efficient and coordinated transcription of phage genes by the T7 RNAp without interference from bacterial RNAp (Savalia *et al.*, 2010). In the solution structure of the complex of Gp2 with a fragment of the β′ jaw domain (*E. coli* RNAp β′ residues 1153–1213), two invariant arginine residues (R56 and R58; R56 and R58 are invariant in 25 and 23, respectively, out of 25 Gp2-like proteins in the EBI database at the time of writing) located in the β3 strand of Gp2 are in close proximity to E1158 and E1188 in the β′ jaw, and thereby contribute to significant ionic interactions across the interface (Fig. 1a, b) (James *et al.*, 2012). Alanine or charge reversal substitutions at either R56 or R58 prevent Gp2 from binding to the RNAp (Cámara *et al.*, 2010). Conversely, RNAp containing a lysine substitution at either E1158 (BR3 *E. coli*) or E1188 (7009 *E. coli*) is resistant to inhibition by Gp2 (Nechaev & Severinov, 1999). Thus, the primary interaction interface between Gp2 and the RNAp involves R56 and R58 in the β3 strand of Gp2 and E1158 and E1188 in the β′ jaw domain of the RNAp.

Wild-type T7 is unable to productively infect *E. coli* 7009 (Chamberlin, 1974; Nechaev & Severinov, 1999; Schmitt *et al.*, 1987). A mutant phage, called T7β, that successfully infects the 7009 *E. coli* strain (which has a mutant RNAp containing the E1188K substitution in the β′ jaw domain), encodes a triple mutant form of Gp2 (harbouring substitutions E24K, F27Y and R56C; hereafter called Gp2^β^) (Chamberlin, 1974; Schmitt *et al.*, 1987). The molecular basis by which E24K, F27Y and R56C substitutions in Gp2 suppress the E1188K mutation in the β′ jaw domain, which prevents wild-type Gp2 from binding to the RNAp, is not known. In the context of the Gp2–β′ jaw domain fragment structure, the E24K and F27Y substitutions are located in the middle and close to the end, respectively, of the loop connecting the β1 and β2 strands in Gp2, i.e. at the opposite side to the β3 strand, which forms the interface with the β′ jaw domain and carries R56 (Fig. 1a). In this study, we established a simple *in vivo* bacterial growth attenuation assay to assess the activity of recombinant Gp2 on *E. coli* RNAp in the absence of T7 infection, to study the molecular basis by which Gp2^β^ inhibits wild-type, 7009 and BR3 *E. coli* RNAp. The results strongly suggest that the E24K and F27Y mutations facilitate the interaction between Gp2^β^ and RNAp when the primary interaction interface between Gp2 and RNAp is compromised. Experiments addressing the loop interconnecting β1 and β2 strands in Gp2 homologues from two T7-like phages that infect *Citrobacter rodentium* further corroborate our results.

## Methods

### 

#### Bacterial strains and plasmids.

Table S1 (available with the online version of this paper) lists the bacterial strains and plasmids used in this study. Plasmid pCS1 : Gp2 and derivatives were constructed by digesting pSW33 : Gp2 (and derivatives) with *Xba*I and *Hin*dIII, and the 0.3 kb fragment containing *gene 2* was cloned into the same sites in pBAD18 (Invitrogen). The coding sequences of Gp2 homologues CR44b protein 13 (CR44b_13) and CR8 protein 16 (CR8_16) were PCR-amplified from phage genomic DNA (kindly provided by Ana Louisa Toribio and Gordan Dougan, The Wellcome Trust Sanger Institute, Cambridge, UK) with primers containing restriction sites for *Nde*I and *Bam*HI and cloned into the same sites in plasmid pET33b+ (Novagen) to generate pAS33 : CR44b_13 and pAS33 : CR8_16, respectively. Similarly, plasmids pAS1 : CR44b_13 and pAS1 : CR8_16 were constructed using the procedure described for pCS1 : Gp2. Mutant forms of T7 Gp2 and CR44b_13 were constructed using the QuikChange Site-Directed PCR Mutagenesis kit (Stratagene) using pCS1 : Gp2, pSW33 : Gp2 or pAS1 : CR44b_13 as a template. T7 Gp2 was overexpressed and purified as described previously; CR44b_13 and CR8_16 were overexpressed and purified exactly as T7 Gp2 (Cámara *et al.*, 2010). Wild-type and mutant forms of the *E. coli* core RNAp were overexpressed from pVS10 and purified by affinity chromatography exactly as described previously (Cámara *et al.*, 2010). The E1188K and E1158K mutant *E. coli* RNAps were constructed by using the QuikChange Site-Directed PCR Mutagenesis kit using pVS10 as the template (Belogurov *et al.*, 2007). Purified proteins were then dialysed into storage buffer [10 mM Tris/HCl (pH 8.0), 50 mM NaCl, 0.1 mM EDTA, 1 mM DTT, 50 % (v/v) glycerol] and aliquots were stored at −20 °C (short-term) and −80 °C (long-term). The sequences of primers used for the cloning and mutagenesis in this study are available from the authors upon request.

#### Bacterial growth attenuation assays.

Seed cultures were grown at 37 °C, shaking at 700 r.p.m. for 6–7 h in a THERMOstar (BMG Labtech) plate incubator by directly inoculating a colony from a freshly transformed Luria agar plate into 200 µl of Luria broth (LB) medium (Difco) containing 100 µg ampicillin ml^−1^ and 0.5 % (w/v) glucose (to prevent leaky expression of Gp2 from the P_BAD_ promoter) into a 96-well microtitre plate (Sterilin). The experimental growth curves were also performed in 96-well microtitre plates in a POLARstar Omega multiwell plate reader (BMG Labtech). The seed cultures were diluted 1 : 100 in a final volume of 200 µl of fresh LB medium containing 100 µg ampicillin ml^−1^ and incubated at 30 °C, shaking at 500 r.p.m. The expression of Gp2 was induced at OD_600_ of ~0.2–0.25 by adding 0.04 % (w/v) arabinose for MG1655, JE1134 and BR3 cultures, and 0.4 % (w/v) arabinose for 7009 cultures. The doubling time (Dt) was calculated from the gradient of the growth curve typically between the OD_600_ corresponding to induction time and OD_600_ 0.7–0.8. The *R*^2^ values in each case were >0.98. At least three biological and technical replicates were performed for each growth curve.

#### Quantitative Western blotting.

**Fig. 2.**
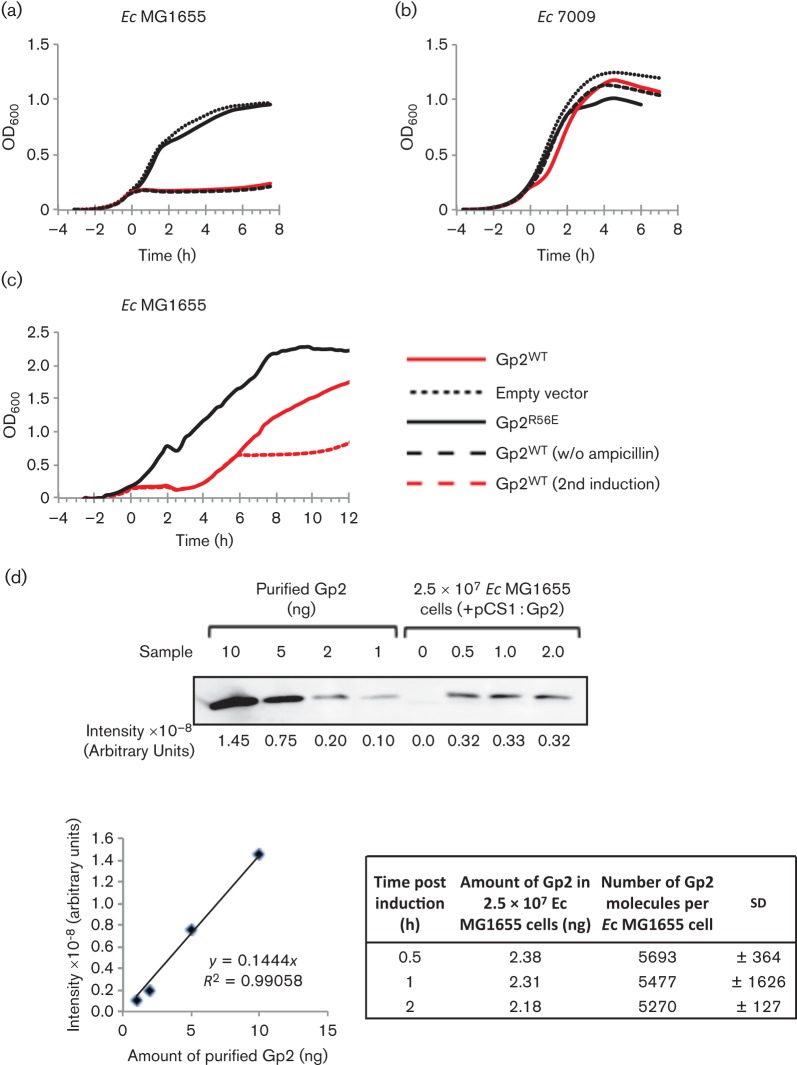
Establishing a simple *in vivo* assay to measure the activity of Gp2 in the absence of T7 phage infection. Growth curves of (a) *E. coli* MG1655 and (b) 7009 cells expressing Gp2 upon induction with arabinose at *t* = 0. The solid red, dotted and solid black lines represent cells harbouring pCS1 : Gp2^WT^, pBAD18 (empty vector) and pCS1 : Gp2^R56E^, respectively; the dashed line represents cells harbouring pCS1 : Gp2^WT^ to which ampicillin was not added to the growth medium. (c) As in (a), except that at *t* = 2 h, the cells were pelleted and resuspended in fresh LB to remove inducer (arabinose) prior to continuation of growth. The dashed red line represents cells that were induced for the second time (see text for details) at *t* = 6 h. (d) Quantification of Gp2 molecules per *E. coli* MG1655 cell at *t* = 0.5, 1 and 2 h post induction of Gp2. Top panel: digitalized image of the Western blot probed with HRP-conjugated anti-His monoclonal antibodies containing purified Gp2 (as a reference standard) and whole-cell protein extracts corresponding to 2.5×10^7^ cells. Bottom-left panel: calibration curve relating signal intensity to the amount of purified Gp2 (in ng). Bottom-right panel: table showing the calculated number of molecules of Gp2 per cell for each time point sampled post induction.

*E. coli* MG1655 cultures were grown in conditions as described above. At 2 h post induction (see text for details), cell samples were taken and the total amount of Gp2 molecules per cell was determined by Western blotting using standard protocols. *E. coli* whole-cell extract (corresponding to 2.5×10^7^ cells) was analysed by SDS-PAGE on gels that were calibrated with known amounts of purified Gp2. Proteins were transferred to a Hybond-ECL nitrocellulose membrane using a Trans-Blot Semi-Dry transfer cell (Bio-Rad). To detect Gp2, anti-His monoclonal antibodies conjugated with horseradish peroxidase (HRP) (Sigma) in combination with the ECL SuperSignal West Femto Chemiluminescent Substrate kit (Pierce) were used. Digital images of the blots were obtained using an LAS-3000 Fuji Imager, and signal quantification and calculations were performed exactly as described by Piper *et al.* (2009). Briefly, the amount of Gp2 (ng) (Fig. 2d, table, column 2) at each time point post induction was estimated from the calibration curve. The number of molecules of Gp2 per cell (Fig. 2d, table, column 3) was determined by first calculating the total number of moles of Gp2 [mass (g)/*M*_r_ (10 000 Da)], multiplying by Avogadro’s constant (6.022×10^23^ mol^−1^) and then by dividing the total number of Gp2 molecules by the number of cells in the sample (i.e. 2.5×10^7^ cells).

#### *In vitro* transcription assays.

These were performed exactly as previously described (Cámara *et al.*, 2010).

#### Efficiency of plaque formation assays.

These were performed exactly as previously described (Cámara *et al.*, 2010; Rokyta *et al.*, 2002).

## Results

### Insights from multiple protein sequence alignment of T7 Gp2 homologues

To determine how amino acids at positions corresponding to T7 Gp2 E24, F27 and R56 compare with corresponding residues in homologous proteins encoded by related phages, we conducted a blast search using standard search parameters and T7 Gp2 as a query sequence. The multiple protein sequence alignment of known Gp2 homologues (in the EBI database, July 2012) was done using cobat (Fig. 1b) (Papadopoulos & Agarwala, 2007). We also built an alignment of the T7 Gp2 target (the β′ jaw fragment region, *E. coli* β′ amino acids 1153–1213) with corresponding sequences from several bacterial species (Fig. 1c). As expected, the amino acid residues R56 and R58 in T7 Gp2 that are essential for the binding to the *E. coli* β′ jaw are identical in 25 and 23, respectively, out of 25 known Gp2 homologues (Fig. 1b). Similarly, amino acid residues E1158 and E1188 display a high degree of conservation only in bacteria infected by phages encoding Gp2 homologues (Fig. 1c). Overall, this observation is consistent with previous structural and biochemical analyses which show that the primary interaction interface between T7 Gp2 and the *E. coli* RNAp involves R56 and R58 in strand β3 of Gp2 and E1158 and E1188 in the β′ jaw domain.

A phenylalanine residue at a position corresponding to F27 of T7 Gp2 is conserved in 18 out of 24 Gp2 homologues, whereas amino acids at a position corresponding to E24 in T7 Gp2 display a significant degree of variation (Fig. 1b). Interestingly, we note that phages CR8, CR44b, K1F and EcoDS1 encode Gp2-like proteins that contain a positively charged amino acid (R15) at the position that corresponds to T7 Gp2 S23, a residue immediately adjacent to E24 that is changed for lysine in Gp2^β^ (Fig. 1b, underlined). From structural models generated for Gp2 homologues from phages CR8 and CR44b and based on the solution structure of the T7 Gp2–β′ jaw domain fragment complex, it is evident that R15 in CR44b_13 and CR8_16 (and by extension also in Gp2 homologues encoded by K1F and EcoDS1) is located in the loop interconnecting β1 and β2 strands of the proteins (Fig. 1d). Thus, it is possible that Gp2 homologues from CR8, CR44b, K1F and EcoDS1 phages, like T7 Gp2^β^, can also inhibit 7009 and/or BR3 RNAp. If true, this would indicate that Gp2 homologues encoded by wild-type CR8, CR44b, K1F and EcoDS1 phages interact with RNAp like the mutant Gp2 encoded by T7β.

### A simple *in vivo* assay measures the activity of Gp2 in the absence of T7 phage infection

To experimentally address the observations derived from multiple sequence alignment and to understand the molecular basis by which the triple amino acid substitutions in Gp2^β^ suppress the E1188K mutation in the β′ jaw domain, we developed a simple *in vivo* assay to determine and compare the ability of wild-type and mutant Gp2 proteins to inhibit *E. coli* RNAp in the absence of T7 infection. In this assay, *gene 2* (which encodes Gp2) is placed under the control of an arabinose-inducible *araBAD* promoter (P_BAD_) in plasmid pCS1 : Gp2 and introduced into *E. coli* strain MG1655. As shown in Fig. 2(a), induction of Gp2 expression upon addition of 0.04 % (w/v) arabinose (at *t* = 0) results in efficient attenuation of bacterial growth. The attenuation of bacterial growth is specific, since it is not observed in wild-type cells transformed with the pCS1 : Gp2^R56E^ plasmid, which encodes a functionally defective Gp2 mutant (Cámara *et al.*, 2010) (Fig. 2a), or in *E. coli* strain 7009 transformed with pCS1 : Gp2 (Fig. 2b). Control experiments established that attenuation of bacterial growth was not caused by a loss of selection due to the inhibition of transcription of the β-lactamase gene from pCS1, since attenuation of bacterial growth upon arabinose induction was observed in the absence of ampicillin (Fig. 2a, b). Additional control experiments, shown in Fig. 2(c), established that recombinant Gp2, when expressed in *E. coli*, acts like a bacteriostatic agent: growth-attenuated *E. coli* MG1655 cells obtained 2 h (i.e. at *t* = 2) after Gp2 induction resumed growth when inoculated into fresh growth medium after removal of the inducer, and stopped growing upon reinduction with arabinose at *t* = 6 h (Fig. 2c). Further analysis of whole-cell extracts from cells obtained at *t* = 2 h after Gp2 induction revealed that the total number of Gp2 molecules per *E. coli* cell is ~5000 molecules (Fig. 2d). Since the total number of RNAp molecules in an exponentially growing *E. coli* cell is estimated to be ~2000–2500 molecules (Ishihama, 1999), it seems that the total number of Gp2 molecules per *E. coli* cell exceeds that of the total number of RNAp molecules per *E. coli* cell by at least twofold under conditions where attenuation of bacterial growth is observed. In summary, the *in vivo* bacterial growth attenuation assay established here can be used to determine and compare the activity of Gp2 mutants to inhibit the *E. coli* RNAp *in vivo* in the absence of T7 phage infection.

### E24K and F27Y substitutions facilitate Gp2 interaction with RNAp when the primary interaction interface is compromised

**Fig. 3.**
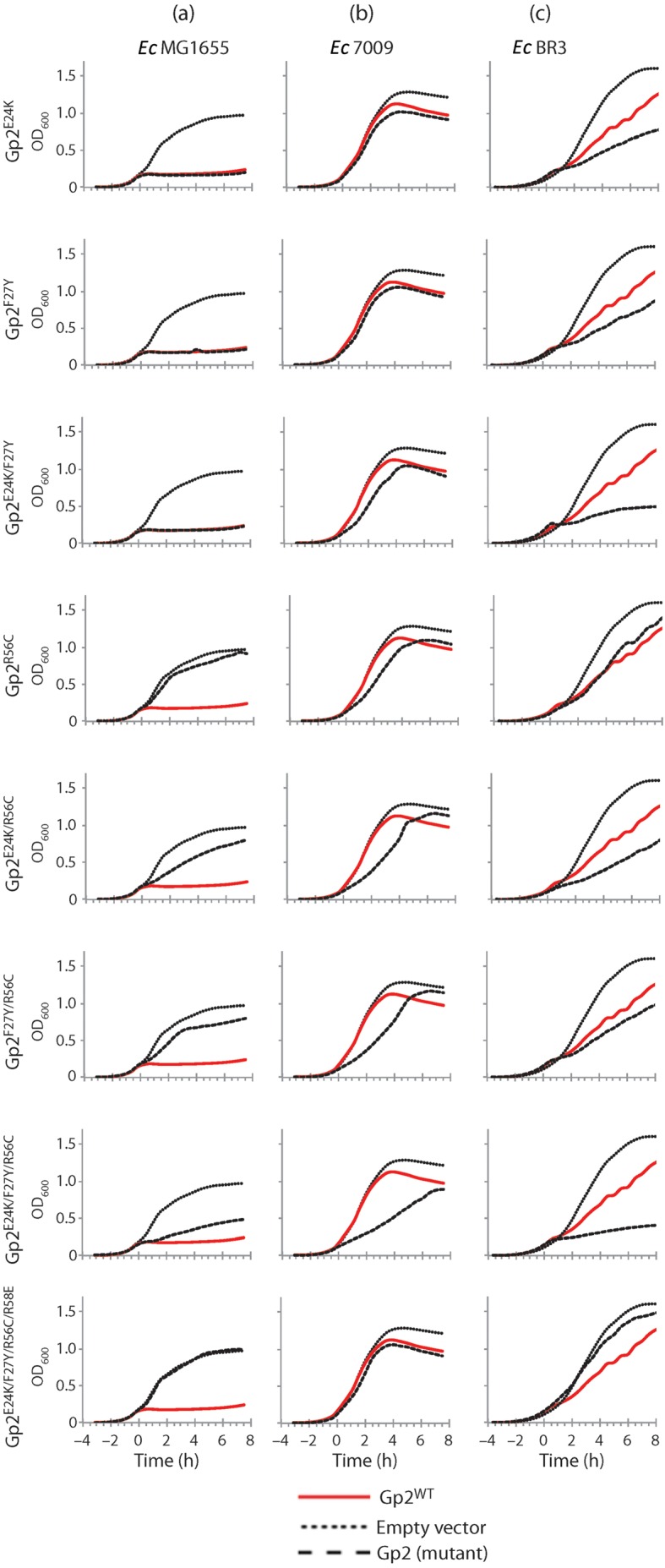
Ability of single, double and triple mutant versions of Gp2 based on Gp2^β^ to attenuate bacterial growth upon induction. Growth curves of (a) *E. coli* MG1655, (b) 7009 and (c) BR3 expressing wild-type Gp2 and Gp2 mutants containing various combinations of mutations based on Gp2^β^. Gp2 expression was induced with arabinose at *t* = 0. In each graph, the solid red, dotted and dashed black lines represent cells harbouring pCS1 : Gp2^WT^, pBAD18 (empty vector) and pCS1 : Gp2 encoding the indicated mutant form of Gp2, respectively. In panels (a–c), the same relevant traces for cells harbouring pCS1 : Gp2^WT^ and pBAD18 (empty vector) were used.

It is not known which one of the three changes (or a combination thereof) restores the binding of Gp2^β^ to the 7009 RNAp. Therefore, we constructed single, double and triple mutant versions of Gp2 based on Gp2^β^ and tested their ability to inhibit the wild-type, 7009 and BR3 *E. coli* RNAp using appropriate strains and the *in vivo* bacterial growth attenuation assay described above (Fig. 2). Consistent with previous results from alanine-scanning mutagenesis (Cámara *et al.*, 2010), single or double amino acid substitutions at E24 (to K) and/or F27 (to Y) attenuated the growth of *E. coli* MG1655 to the same degree, as did the wild-type Gp2. On the other hand, expression of Gp2^R56C^ did not significantly affect the growth rate (expressed as Dt in Table 1) as compared with cells harbouring the empty plasmid vector (pBAD18), indicating that the R56C substitution strongly affected the interaction with the wild-type RNAp. However, the Dt of cells expressing double mutant Gp2 variants containing the R56C mutation in combination with either the E24K or the F27Y mutation (i.e. Gp2^E24K/R56C^ and Gp2^F27Y/R56C^) was detectably increased (by ~4.1- and ~1.8-fold, respectively) compared with the growth rate of cells harbouring the empty plasmid vector (Fig. 3a, Table 1). Although compared with wild-type Gp2, Gp2^E24K^, Gp2^F27Y^ and Gp2^E24K/F27Y^ mutants, the Gp2^E24K/R56C^ and Gp2^F27Y/R56C^ mutants attenuated the growth of MG1655 *E. coli* cells relatively weakly (Fig. 3a, Table 1), the effect was highly reproducible. When present together, the E24K and F27Y mutations further improved the ability of Gp2^R56C^ (i.e. Gp2^β^) to attenuate wild-type *E. coli* growth (Fig. 3a), and the Dt of cells expressing Gp2^β^ was increased ~5.3-fold compared with that of cells harbouring the empty plasmid vector (Table 1). In summary, it appears that mutations in amino acids residues in and surrounding the loop interconnecting β1 and β2 strands in Gp2 (i.e. E24K and F27Y) can compensate for mutations in Gp2 (i.e. R56C) that compromise (but not abolish; see below) the interaction interface between Gp2 and the β′ jaw domain. Based on Dt values calculated for MG1655 *E. coli* cells after induction of Gp2^E24K/R56C^, Gp2^F27Y/R56C^ and Gp2^β^ (Table 1), it seems that the charge reversal substitution at E24 (i.e. E24K) in the loop interconnecting the β1 and β2 strands in Gp2 rather than the F27Y mutation at the beginning of the β2 strand is more important in enabling the interaction between Gp2 and the RNAp when the primary interaction interface with the β′ jaw is compromised by the R56C substitution. However, since a glutamate substitution at position R58 in the context of Gp2^β^ effectively abolishes the ability of Gp2^β^ to attenuate the growth of wild-type *E. coli*, it would seem that an interaction interface between the β3 strand and β′ jaw domain still persists in the Gp2^β^–RNAp complex (Fig. 3a). Experiments with the 7009 and BR3 *E. coli* strains, which encode Gp2-resistant forms of RNAp (see Introduction), further corroborated this view: even though the Dt of 7009 and BR3 *E. coli* cells, when compared with the MG1655 *E. coli* cells, is relatively unaffected by overproduction of wild-type Gp2, Gp2^β^ increases the Dt of 7009 and BR3 *E. coli* cells by ~2.6- and 2.2-fold, respectively, compared with corresponding cells expressing wild-type Gp2 (Fig. 3b, c, Table 1). Interestingly, in the context of the BR3 *E. coli* cells, expression of Gp2^E24K/F27Y^ attenuates growth as efficiently as that of Gp2^β^ (Fig. 3c, Table 1). In both cases, the R58E substitution abolishes the ability of Gp2^β^ to attenuate cell growth (Fig. 3b, c). Overall, the results strongly suggest that the E24K and F27Y substitutions facilitate an interaction between Gp2 and RNAp when the primary interaction interface in the β3 strand is compromised.

**Table 1.** Dt values for *E. coli* MG1655, 7009 and BR3 cells expressing wild-type Gp2 and mutant Gp2 containing different combinations of point mutations based on Gp2^β^ nd, Not determined.

Gp2	*Ec* MG1655 Dt* (min)±sd†	FI‡	*Ec* 7009 Dt (min)±sd	FI§	*Ec* BR3 Dt (min)±sd	FI||
Empty vector	53±5	1.0	54±3	nd	66±1	nd
Wild-type	nd	nd	58±1	1.0	117±6	1.0
E24K	nd	nd	57±4	1.0	179±6	1.5
F27Y	nd	nd	54±3	1.0	176±10	1.5
E24K/F27Y	nd	nd	57±2	1.0	249±7	2.1
R56C	64±1	1.2	65±2	1.2	102±4	0.9
E24K/R56C	221±8	4.1	85±3	1.6	166±9	1.4
F27Y/R56C	93±11	1.8	83±5	1.6	151±2	1.3
E24K/F27Y/R56C	280±48	5.3	142±5	2.6	262±1	2.2
Gp2^β^+R58E	52±1	1.0	59±8	1.1	73±5	0.6

* Dt (doubling time) in LB at 30 °C in microtitre plates.

† sd is based on at least three independent measurements for each strain/plasmid combination.

‡ Fold increase in Dt with respect to MG1655 *E. coli* cells containing the empty vector.

§ Fold increase in Dt with respect to 7009 *E. coli* cells expressing wild-type Gp2.

||Fold increase in Dt with respect to BR3 *E. coli* cells expressing wild-type Gp2.

### T7 Gp2 amino acids 14–59 are sufficient to inhibit the *E. coli* RNAp

**Fig. 4.**
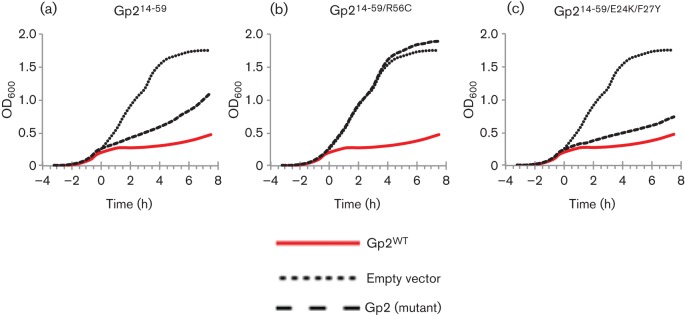
Amino acids 14–59 of Gp2 are sufficient to inhibit the *E. coli* RNAp. Growth curves of *E. coli* MG1655 cells in which the expression of (a) Gp2^14–59^, (b) Gp2^14–59/R56C^ and (c) Gp2^14–59/E24K/F27Y^ was induced at *t* = 0 (dashed lines). In each graph, the solid red and dotted lines represent cells harbouring pCS1 : Gp2^WT^ and pBAD18 (empty vector), respectively.

It is evident from the alignment (Fig. 1b) that the highest degree of sequence conservation occurs between residues corresponding to T7 Gp2 amino acids 14–59. Therefore, it is possible that amino acids outside this region are dispensable for activity. We used the *in vivo* bacterial growth attenuation assay to determine whether the truncated form of Gp2 (Gp2^14–59^) is functionally active. Results shown in Fig. 4(a) and Table 2 reveal that expression of Gp2^14–59^ detectably attenuates MG1655 *E. coli* cell growth, though not as efficiently as full-length Gp2 expression. The Dt of MG1655 cells expressing Gp2^14–59^ is increased ~4.9-fold when compared with MG1655 *E. coli* cells harbouring the empty plasmid vector (Table 2). Since further N- and C-terminal truncations (e.g. Gp2^16–59^, Gp2^14–58^ and Gp2^14–55^) resulted in proteins unable to affect cell growth rate (data not shown), the results suggest that a minimal functional region of T7 Gp2 comprises amino acids 14–59. The attenuation of cell growth by Gp2^14–59^ is specific, since no growth attenuation is detected in the context of the R56C mutation (Fig. 4b). However, the E24K and F27Y mutations clearly improve the efficiency by which Gp2^14–59^ attenuates MG1655 cell growth (Fig. 4c). The Dt of MG1655 *E. coli* cells expressing Gp2^14–59^ carrying the E24K/F27Y double substitution is increased ~6.9-fold compared with that of cells harbouring an empty plasmid vector (Table 2). This result indicates that E24K and F27Y substitutions improve the binding of Gp2 to the RNAp even if the primary interaction interface with the β′ jaw domain is intact but the binding affinity is attenuated by N- and C-terminal deletion of evolutionarily variable segments.

**Table 2.** Dt values for *E. coli* strain MG1655 expressing wild-type and mutant versions of Gp2^14–59^ nd, Not determined.

Gp2	*Ec* MG1655 Dt* (min)±sd†	FI‡
Empty vector	49±1	1.0
Wild-type	nd	nd
Gp2^14–59^	239±16	4.9
Gp2^14–59^ (R56C)	57±2	1.2
Gp2^14–59^ (E24K/F27Y)	338±25	6.9

* Dt (doubling time) in LB at 30 °C in microtitre plates.

† sd is based on at least three independent measurements for each strain/plasmid combination.

‡ Fold increase in Dt with respect to MG1655 *E. coli* cells containing the empty vector.

### Gp2 homologues from phages that infect *C. rodentium* inhibit *E. coli* RNAp even if the interaction with the β′ jaw is compromised

To obtain further experimental evidence that E24K and F27Y substitutions in Gp2 facilitate the interaction of Gp2 with RNAp when the primary interaction interface with the β′ jaw domain is compromised, we conducted experiments with Gp2 homologues, proteins 13 and 16 from phages CR44b and CR8 (CR44b_13 and CR8_16), which infect *C. rodentium*, a model animal bacterial pathogen used to study human infections by enteropathogenic and enterohaemorrhagic *E. coli*. Initially, we wanted to determine whether recombinant CR44b_13 and CR8_16 can complement a T7 mutant phage (T7*2am64*) harbouring a mutation in *gene 2* (Burck & Miller, 1978). T7*2am64* infections of wild-type *E. coli* BL21 cells are not productive, since Gp2 function is essential for phage development. However, *E. coli* BL21 cells containing pSW33 : Gp2^WT^ (which allows expression of Gp2 from a T7 RNAp-dependent promoter) are successfully infected by T7*2am64*, as judged by the efficiency of plaque formation, which gave an efficiency of plating (EOP) value of 1.0±0.18. EOP was measured using the following equation: EOP = (number of T7*2am64* plaques on strain+test plasmid)/(number of T7*2am64* plaques on strain+pSW33 : Gp2^WT^). In contrast, the EOP was significantly reduced (to 0±0.01) on lawns of *E. coli* BL21 cells containing pSW33 : Gp2^R56E^, which encodes a functionally defective Gp2 mutant. When plasmids encoding recombinant CR44b_13 (pAS33 : CR44b_13) were transformed into *E. coli* BL21, T7*2am64* formed plaques, albeit at 40 % reduced efficiency compared with *E. coli* BL21 cells harbouring pSW33 : Gp2^WT^ (EOP of 0.61±0.02). However, T7*2am64* was able to form plaques equally well on *E. coli* BL21 cells harbouring a plasmid encoding CR8_16 (pAS33 : CR8_16) (EOP of 1.01±0.21) and wild-type Gp2 (pSW33 : Gp2^WT^). Thus, we conclude that CR44b protein 13 and CR8 protein 16 can partially and fully, respectively, substitute for T7 Gp2 during T7 infection.

**Fig. 5.**
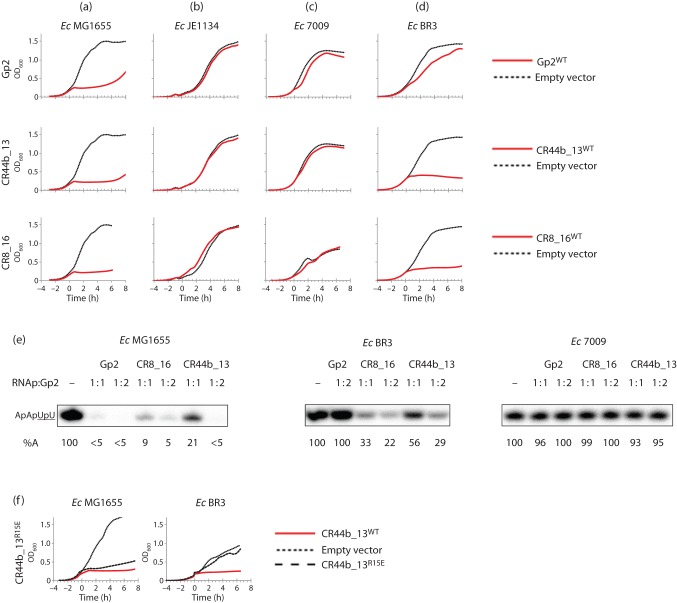
Experiments with Gp2-like proteins from *C. rodentium* phages CR44b and CR8. Growth curves of (a) *E. coli* MG1655, (b) JE1134, (c) 7009 and (d) BR3 cells in which the expression of T7 Gp2 and Gp2-like proteins CR44b_13 and CR8_16 was induced at *t* = 0 (solid red lines). In each graph, the dotted line represents cells harbouring pBAD18 (empty vector). (e) Autoradiograph of a 20 % (w/v) denaturing gel showing synthesis of the transcript ApApUpU (the ^32^P-labelled nucleotides underlined) from the *lac*UV5 promoter by the σ^70^-containing RNAp in the presence of Gp2, CR44b_13 and CR8_16. The molar ratio of RNAP to Gp2/Gp2-like proteins is indicated above each lane. The percentage of ApApUpU transcript synthesized (%A) by the RNAp is calculated relative to the reaction without Gp2 and is indicated at the bottom of each lane. (f) Growth curves of *E. coli* MG1655 (left) and BR3 (right) cells expressing CR44b_13 and harbouring the R15E amino acid substitution. Induction with arabinose was performed at *t* = 0.

Next, we conducted a growth attenuation assay to establish whether CR44b_13 and CR8_ 16, like T7 Gp2, bind to the β′ jaw domain of the *E. coli* RNAp. As shown in Fig. 5(a), induction of CR44b_13 and CR8_16 expression with 0.04 % (w/v) arabinose from pAS1 : CR44b_13 and pAS1 : CR8_16, respectively, in wild-type *E. coli* MG1655, resulted in efficient growth attenuation. However, no growth attenuation of *E. coli* strain JE1134, which encodes a β′ subunit with residues 1149–1190 deleted (Fig. 5b), was observed. We next determined whether CR44b_13 and CR8_16 can inhibit *E. coli* RNAp harbouring either the E1158K (i.e. found in BR3 *E. coli* strain) or E1188K (i.e. found in 7009 *E. coli* strain) mutations, as suggested by sequence analysis (above). As shown in Fig. 5(c), whereas the growth of *E. coli* strain 7009 was not detectably attenuated by either CR44b_13 or CR8_16, the growth of *E. coli* strain BR3 was efficiently attenuated by both phage proteins (Fig. 5d). *In vitro* transcription assays, which report the ability of T7 Gp2 CR44b_13 and CR8_16 proteins to inhibit the synthesis of the tetranucleotide RNA product (ApApUpU) from the *lac*UV5 promoter by the RNAp containing σ^70^, with purified wild-type, BR3 and 7009 RNAp and T7 Gp2, confirmed the *in vivo* results (Fig. 5e).

If a charge reversal substitution (E24K) in T7 Gp2^β^ indeed facilitates binding to the RNAp when the primary contact with the β′ jaw domain is weakened (Fig. 3), then R15 of CR44b_13 should be important for attenuation of BR3 *E. coli* growth. Results shown in Fig. 5(f) confirm that this is indeed the case: the mutant variant of CR44b_13 harbouring the R15E mutation fails to attenuate the growth of *E. coli* strain BR3 upon induction. However, the R15E mutation has no detectable effect on the ability of CR44b_13 Gp2 to attenuate the growth of wild-type *E. coli*. Overall, results with Gp2-like proteins encoded by *C. rodentium* phages provide further support for the view that the molecular basis by which Gp2^β^ is able to inhibit the 7009 RNAp is that E24K and F27Y substitutions facilitate an interaction between Gp2 and RNAp when the primary interaction interface is compromised.

## Discussion

T7 Gp2 binds tightly to and potently inhibits *E. coli* RNAp. Previous biochemical and structural studies have shown that amino acid residues in the β3 strand of Gp2 contribute to the primary interaction interface with the β′ jaw domain of the RNAp. Results from the current study show that substitutions in amino acid residues of Gp2 located in the region surrounding and including the loop interconnecting the β1 and β2 strands, and, therefore, located on the opposite side to the β3 strand, can compensate for amino acid substitutions in Gp2 and/or the β′ jaw domain that compromise the primary interaction interface between Gp2 and the RNAp. This seems to constitute the molecular basis by which T7β (encoding Gp2^β^ harbouring the E24K, F27Y and R56C substitutions) can successfully infect the 7009 *E. coli* strain (harbouring the E1188K mutation) that is resistant to infection by wild-type T7 phage.

**Fig. 6.**
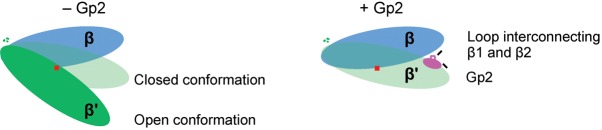
Gp2 induces RNAp to adopt the ‘closed state’ conformation. Cartoon showing how the region including and surrounding the β1 and β2 strands in Gp2 could contribute to inducing the closed state conformation in the RNAp.

Since the E24K and F27Y substitutions allow (1) the functionally defective Gp2 mutant with the R56C substitution effectively to attenuate wild-type *E. coli* growth (Fig. 3a, Table 1) and (2) detectably improve the ability of the truncated Gp2 form, Gp2^14–59^, to attenuate wild-type *E. coli* growth (Fig. 4, Table 2), it is possible that the region of Gp2 surrounding and including the loop interconnecting the β1 and β2 strands contributes to an ‘auxiliary’ interaction interface between Gp2 and RNAp and contributes to the mechanism by which Gp2 efficiently inhibits RPo formation by the RNAp: the overall architecture of RNAp is reminiscent of a ‘crab claw’. The β and β′ subunits form the two ‘pincers’ of the claw and define the DBC and dwDBC (see Introduction). During RPo formation access to the DBC and dwDBC is controlled by large-scale movements of the β′ subunit (Mekler *et al.*, 2002). Thus, with respect to accessibility of the DBC, RNAp can exist in a so-called ‘closed state’ (in which the width of the DBC is insufficient to allow access of double-stranded DNA) or in an ‘open state’ (in which the DBC is sufficiently wide to allow access of double-stranded DNA) (Chakraborty *et al.*, 2012). The open state is required for RPo formation, and after loading and unwinding of DNA to form the transcription bubble, the RNAp converts into the closed state. Furthermore, the binding of dwDNA to the dwDBC and the concomitant displacement of the σ^70^ domain, known as region 1.1, from the catalytic cleft are the minimal pre-requisites for efficient and stable RPo formation at promoters utilized by the RNAp containing the σ^70^ factor (Mekler *et al.*, 2002). Gp2 seems to employ a three-pronged strategy to inhibit RPo formation by (i) sterically antagonizing the interaction between dwDNA and the β′ jaw domain, (ii) preventing obligatory displacement of σ^70^ region 1.1, and (iii) inducing RNAp to adopt the closed state, thereby restricting DNA access to the DBC and dwDBC (Cámara *et al.*, 2010; Chakraborty *et al.*, 2012; James *et al.*, 2012; Mekler *et al.*, 2011). Thus, the interface between Gp2 and the RNAp could consist of at least two interaction interfaces: a primary one with the β′ jaw and one or more auxiliary interaction interfaces. We hypothesize that these interfaces collectively contribute to the high affinity between Gp2 and the RNAp and orchestrate the multipronged mechanism of transcription inhibition by Gp2. Specifically, we envisage a model in which, when Gp2 is bound to the jaw domain on the β′ pincer, the region surrounding and including the loop interconnecting the β1 and β2 of Gp2 interacts with the β pincer (which is located directly across from the β′ jaw on other side of the DBC) and thereby ‘locks’ the RNAp in the closed state, leading to very efficient inhibition of RPo formation (Fig. 6). This mechanism of inhibition of RPo formation by Gp2 is analogous to how some antibiotics (such as myxopyronin, corallopyronin and ripostatin) and the non-DNA-binding transcription factor DksA inhibit RPo formation by allosterically inducing RNAp to adopt the closed state (Chakraborty *et al.*, 2012; Rutherford *et al.*, 2009). However, in terms of interaction with the RNAp, Gp2 seems to be analogous to the transcription anti-termination factor RfaH, which binds to β′ clamp helices and a β gate loop, which are located directly across from each other on the two sides of the DBC and thereby lock the RNAp in the closed state to ensure transcription processivity, reducing pausing and termination (Sevostyanova *et al.*, 2011). Experiments to verify the model in which Gp2 simultaneously interacts with the β and β′ pincers to inhibit RPo formation are currently in progress.
